# The Case of Mr. Whitman

**Published:** 1848-02

**Authors:** E. Clark


11. *The case of Mr. Whitman.* (From the Portland Argus.)

The case of the late lamented Rev. Jason Whitman, who died in this city, of pleurisy, has excited more than usual interest. It was known to his friends that from early infancy he had suffered from a peculiar cough, and copious muco-purulent expectoration. He seemed predisposed to pleurisy, from which he had suffered several attacks.

The peculiar symptoms of his case, led him to suspect there might be some malformation, which must preclude the hope of perfect health; and he expressed the wish, that, at his decease, a post-mortem examination might be made. It is believed that a short description of this wonderful case may be interesting to your readers.

The examination was made by myself, in the presence of Drs. S. Weed, A. Rea, Wm. Wood, J. T. Gilman, J. T. G. Daveis, M. Dodge, and L. Fitch; and Wm. Willis and Martin Gore, Esqs.

When the lungs were exposed, they were observed to be united by old firm adhesions, laterally to the pleura covering the ribs—and below to the diaphragm. These adhesions gave proof of previous attacks of inflammation. At the inferior part of the left lung was a deposit of pus—the result of the last attack of disease. This was contained in a small sac formed by the pleura of the lungs on the one side, and of the ribs on the other side, and the only part in both lungs not previously bound to contiguous parts by strong adhesions.

The two lobes of the left lung filled the left cavity of the thorax. The heart was in the right cavity, having its apex or small end inclined to the right, and not towards the left side, as in the natural position. The right lung has three lobes; in this case there were only two; the place of the third being occupied by the heart.

The liver was in the left side, of nearly natural size, and perfectly reversed from—united to the left, and not to the right hypochondrium. The position of the stomach was reversed, the small end to the left, and the large end towards the right side. The spleen and larger end of the stomach occupied the place usually assigned to the liver. The large intestines, always found on the right, were here situated in the left side of the abdomen, and passing from left to right, instead of their natural reversed direction, from right to left. The sigmoid flexure, always in the left, was in this case found in the right side. All organs of the abdomen were healthy, and their form perfect, but reversed. The great omentum, or caul, was wanting. The other organs examined were natural in form and situation.

It was matter of regret that time did not allow us to pursue the examination of the arteries, as peculiarities in his pulse had been observed during life, which might perhaps have found a solution. So strange a natural position of so many important organs was never before seen by any of the physicians who witnessed the examination.

Yours, E. Clark.

